# P193: Nasal carriage of methicillin resistant Staphylococcus aureus in staff of the surgical services of CHU Sylvanus Olympio Lome-Togo

**DOI:** 10.1186/2047-2994-2-S1-P193

**Published:** 2013-06-20

**Authors:** I Watéba, M Salou, D Ekouevi, D Dosseh, S Dossim, SD Tigossou, AY Dagnra, M Prince-David

**Affiliations:** 1 Service Respiratory and Infectious Diseases, CHU Sylvanus Olympio, Lomé, Togo; 2 Laboratory of Microbiology, CHU Sylvanus Olympio, Lomé, Togo; 3 Department of Basic Sciences and Public Health, Togo; 4 Department of General Surgery, CHU Sylvanus Olympio, Lomé, Togo

## Introduction

Methicillin resistant Staphylococcus aureus (MRSA) is a major public health problem found in nosocomial infections. However, one of the possible sites of carriage in health care workers is the nasal cavity.

## Objectives

To estimate the prevalence of nasal carriage of MRSA in the surgical staff of the Hospital of Lomé Sylvanus Olympio.

## Methods

This cross-sectional study was conducted from 1 July 2011 to 31 October 2011. The samples were obtained by nasal swab of healthcare workers in the surgical services of the hospitak namely the central surgical, trauma, pediatric surgery, visceral surgery and surgical intensive care wards. These samples were inoculated on Chapman agar. The identification of isolated staphylococci was completed by the agglutination test using Pastorex Staph Plus kit. The susceptibility of S. aureus was performed according to the recommendations of the French Society for Microbiology. The resistance to methicillin was highlighted by the use of cefoxitin disks and oxacillin.

## Results

Ninety-five (95) people participated in the study, 17 (18%) were MRSA positive. Carriage rates were distributed as follows: Traumatology 5/11, surgical center 6/25, visceral surgery 2/14, pediatric surgery 2/16, surgical ICU 3/29. The nurses of the first two services and doctors of visceral surgery were the most colonized with MRSA. MRSA isolates were resistant to aminoglycosides: kanamycin (88%), tobramycin (82%), Gentamicin (64%), quinolone: pefloxacin (70%).,MRSA strains were more susceptible to macrolides and related drugs: Erythromycin (76%), Lincomycin (82%), pristinamycin (100%). No MRSA were resistant to vancomycin.

## Conclusion

This study confirmed a high carriage rate of MRSA in the surgical staff of the Hospital Sylvanus Olympio and should encourage the development of appropriate preventive health measures such as the application of mupirocin in the context of fight against infections.

## Disclosure of interest

None declared

